# Systematic Approach for Remote Sensing of Historical Conflict Landscapes with UAV-Based Laserscanning

**DOI:** 10.3390/s22010217

**Published:** 2021-12-29

**Authors:** Marcel Storch, Thomas Jarmer, Mirjam Adam, Norbert de Lange

**Affiliations:** 1 Research Group Environmental Informatics and Municipal Planning, Institute of Computer Science, Osnabrück University, 49074 Osnabrück, Germany; ndelange@uni-osnabrueck.de; 2 Remote Sensing Group, Institute of Computer Science, Osnabrück University, 49074 Osnabrück, Germany; thomas.jarmer@uni-osnabrueck.de; 3 Interdisciplinary Research Group on Conflict Landscapes, Osnabrück University, 49074 Osnabrück, Germany; miadam@uni-osnabrueck.de; 4 Modern History and Historical Migration Research, Osnabrück University, 49074 Osnabrück, Germany

**Keywords:** UAV, LiDAR, historical conflict landscapes, terrain anomalies, flight parameters, influencing factors, filter algorithms, ground monuments, heritage sites

## Abstract

In order to locate historical traces, drone-based Laserscanning has become increasingly popular in archaeological prospection and historical conflict landscapes research. The low resolution of aircraft-based Laserscanning is not suitable for small-scale detailed analysis so that high-resolution UAV-based LiDAR data are required. However, many of the existing studies lack a systematic approach to UAV-LiDAR data acquisition and point cloud filtering. We use this methodology to detect anthropogenic terrain anomalies. In this study, we systematically investigated different influencing factors on UAV-LiDAR data acquisition. The flight parameters speed and altitude above ground were systematically varied. In addition, different vegetation cover and seasonal acquisition times were compared, and we evaluated three different types of filter algorithms to separate ground from non-ground. It could be seen from our experiments that for the detection of subsurface anomalies in treeless open terrain, higher flight speeds like 6 m/s were feasible. Regarding the flight altitude, we recommend an altitude of 50–75 m above ground. At higher flight altitudes of 100–120 m above ground, there is the risk that terrain characteristics smaller than 50 cm will be missed. Areas covered with deciduous forest should only be surveyed during leaf-off season. In the presence of low-level vegetation (small bushes and shrubs with a height of up to 2 m), it turned out that the morphological filter was the most suitable. In tree-covered areas with total absence of near ground vegetation, however, the choice of filter algorithm plays only a subordinate role, especially during winter where the resulting ground point densities have a percentage deviation of less than 6% from each other.

## 1. Introduction

### 1.1. Remote Sensing as a Method to Analyze Conflict Landscapes

‘Conflict landscapes’ have become a central matter within historical and archaeological sciences in discussing sites of historical events which are related to acts of great violence—such as battlefields—which therefore have left their mark on the materiality as well as the immateriality of these landscapes [1]. Concerning the scientific research of these sometimes strongly transformed sites—due to different factors–a wide interdisciplinary scientific work field is required to detect these seemingly invisible traces of the past [2,3,4,5]. Thus, mapping historical features and remains is very important to get more information on the historical events than sources like files, photographs, oral history, etc., alone could provide. The remains differ with respect to the purpose and manner in which they were created, the decade of creation and the extent to which they have been subject to erosional or backfilling processes over time up until the present.

In order to locate historical traces and remains, fieldwork is one approach in historical and archaeological science. Even though on-site prospecting generally generates highly reliable and detailed information, it is a very labour-intensive and time-consuming data collection method. It is therefore neither possible to investigate areas of several hectares within a short period of time (e.g., one day), nor can the analysis be frequently repeated (e.g., every month) if the most recent changes in the landscape on-site such as the destruction of historical remains need to be documented. One way to overcome these limitations is to use remote sensing technology [5]. Remote sensing generally means gathering information about the characteristics of the landscape by measuring the reflected irradiance from above. A variety of remote sensing methods exists that are usually divided into passive and active methods. Passive systems like multispectral or hyperspectral sensors measure the solar radiation reflected from the earth’s surface and objects and vegetation on the surface and are thus highly dependent on the weather and light conditions during data acquisition. In addition, these techniques cannot capture the geometry of the earth’s surface in vegetated areas because the vegetation canopy can prevent the irradiation from reaching the ground [6,7].

Active remote sensing methods can, in contrast, generate their own signals at a specific wavelength, which makes them insensitive to the environmental parameters mentioned above. In addition, they can penetrate the vegetation canopy and thus provide information about sub-canopy features, which is of enormous relevance for archaeological and historical research [8], as many of the anomalies that can still be detected are often located in vegetated areas, which are therefore not as strongly transformed as anomalies in the open field. Vegetation cover can help preserving historical remains as the terrain anomalies are less exposed to erosion than on bare ground [9,10]. This allows to detect some of the seemingly invisible traces of the past which are especially important in contexts of violent events where transformation was intentionally induced in order to erase the evidence of such events (e.g., the concealment of killing sites or locations of concentration camps). Due to these facts, the active remote sensing technologies SAR (Synthetic Aperture Radar) and LiDAR (Light Detection And Ranging, also known as laser scanning) have emerged as important data acquisition methods. By analyzing the backscattered amplitude and phase of a SAR system, information about the properties of the ground surface can be obtained. For instance, topographic information can be generated from multiple SAR images by interferometric analysis. Therefore, many airborne and spaceborne SAR systems were used for archaeological prospection and cultural heritage monitoring in the past years (see for example Luo et al. [11] for a comprehensive overview). However, the detectability of surface structures in SAR images can to some extent be limited because of the speckling effect [12], whereas three-dimensional scanning of historically relevant landscapes with the use of laser scanning (LiDAR) can usually be used to perform even more fine-scale analyses, especially when the scanner is not mounted on an aircraft or satellite but on a drone.

Many parameters can influence drone-based LiDAR data acquisition. The aim of this paper is a systematic approach for high-resolution drone-based laser scanning of historical conflict landscapes in order to provide the reader with information and guidance on how to best conduct such surveys to reveal terrain anomalies that indicate historical traces and remains. In the following Section 1.2, related work and literature with regard to the use of LiDAR remote sensing within historical conflict landscapes research is presented. Deduced from the literature review, the Section 1.3 then further specifies the objectives and the approach of this study.

### 1.2. LiDAR Remote Sensing within Historical Conflict Landscapes Research–State of the Art

For instance, Ronchi et al. [13] showed that LiDAR outperforms RGB or multispectral approaches in the context of terrain anomaly identification. A LiDAR system generally consists of a single-wavelength laser that typically operates at a near-infrared wavelength. The scanner periodically sends out light pulses and gathers information about the surface by analyzing the returned echoes. Depending on the size of the research area and the data accuracy requirements resulting from the research questions, a laser scanner device can be mounted on one of the following platforms: Satellite (space-born LiDAR), plane (airborne laser scanning, ALS), drone (unmanned aerial vehicles, UAV-LiDAR, sometimes also the abbreviation RPA, Remotely Piloted Aircraft, is used) or statically mounted on a tripod (terrestrial laser scanning, TLS) [11].

Due to the coarse spatial resolution of *space-borne* LiDAR systems, which are characterized by footprint diameters of several meters, they are of limited use for small-scale archaeological or cultural heritage issues [11,14] but are more suitable for large-scale applications in bathymetry [15] or forestry [16,17].

Contrary to satellite-based laser scanning, the term *airborne* LiDAR usually refers to LiDAR data acquired by plane. Those systems are capable of achieving much higher accuracies with laser footprint diameters on the surface in the order of a few decimeters [14]. Often airborne LiDAR data are periodically acquired and made available to the public free of charge by local authorities [7,18]. During the last years, the use of airborne LiDAR for detecting and documenting historical remains has thus been investigated by several authors. ALS has therefore become a widely used method for terrain anomaly detection in the context of cultural heritage, for example, detecting ancient mound or pit structures [7,19,20,21] or cave entries [22,23]. It is also frequently used in research on conflict-related sites to map remains of battlefields of World War I [24,25] and World War II [26,27,28].

Even though ALS has proven its advantages and usefulness in this context, this method embodies limitations in terms of accuracy and spatial resolution, i.e., point density. In general, typical ALS systems are able to achieve laser shot densities on the order of approx. 5–25 laser shots per square meter [11,14,27,28,29]. It should be noted that the actual received return density can be higher, since a single emitted laser shot may generate multiple return signals, particularly in vegetated areas. On the downside, the resulting ground point density may be lower, since not every laser shot might eventually reach the ground [14]. Especially in wooded areas, this can result in small terrain anomalies (with horizontal diameters of a few meters) and shallow terrain anomalies (with vertical height differences of one meter or less) not being detected or being detected very poorly [19,21]. The average point density and point spacing of *terrestrial* LiDAR scans, however, are much higher than those of airborne systems. These properties allow for very fine-scale site-level analysis [11,30], but working with ground-based systems is time-consuming and not possible in terrain that is difficult to access, also due to the cumbersome and heavy TLS device.

*Drone-based (UAV)* laser scanning is a relatively new technology that has become increasingly popular in archaeological prospection and historical research in recent years as it offsets the disadvantages of all other platforms mentioned above. Since UAVs fly at lower altitudes above ground and at lower speed than aircraft, UAV-LiDAR systems can achieve point densities more than ten times higher than those of airplane-based systems. Digital terrain models (DTM) generated from such data can therefore offer great added value when surveying historically relevant landscapes to detect and document historical traces and remains [31,32,33,34,35]. Since this technology is relatively new, there are several unanswered questions about its systematic use for archaeological prospection and cultural heritage monitoring. For example, it could be that flight parameters are set unfavorably with respect to the conditions in the particular study area, so that certain terrain anomalies cannot be detected by the system. However, the flexibility of UAV-LiDAR, e.g., the possibility of adapting the flight planning individually and adjusting the flight parameters depending on the application and research question, is one of the advantages of this technique compared to airplane-based LiDAR [13,31,35].

### 1.3. Objectives of This Article

Many of the existing studies lack a systematic approach to UAV-LiDAR data acquisition for archaeological prospection. For instance, Risbøl et al. [34] compared ALS and UAV-LiDAR data of different point densities and showed the improvements of high-resolution data sets but did not investigate the effect of different UAV flight parameters or the difference between leaf-on and leaf-off season. The latter is partially discussed by Zhou et al. [35], although this study also does not systematically vary individual flight parameters in a study area. Mesas-Carrascosa et al. [36] evaluated the influence of the UAV altitude above ground, but they used RGB optical imagery for the generation of orthomosaics. A more detailed description of UAV flight planning in the context of LiDAR can be found in Khan et al. [32], but another particular aspect is not comprehensively addressed in both [32] and the previously mentioned studies: *filtering* of UAV-LiDAR point clouds, which means classifying LiDAR data points into ground vs. non-ground to generate digital terrain models (DTM). Many different algorithms exist for this purpose [37,38,39], but the choice of the filter algorithm used and the appropriate DTM generation procedure are often not in focus [32,34,35]. However, these are important steps in using UAV-LiDAR data for historical or archaeological interpretations. The filtering algorithm and further processing steps can have decisive influence on the discrimination of archaeological features in the resulting DTM [11,14]. This is for example addressed by Štular et al. [40,41] in the context of ALS data, but not explicitly for the case of high-resolution UAV-LiDAR data.

Therefore, the aim of this article is to examine the factors influencing UAV-LiDAR data acquisition in different types of typical historical conflict landscapes. Although these each have individual significance due to their respective historical context, we specifically select the study areas for this paper in order to be able to systematically vary and investigate the influencing factors from the perspective of UAV-LiDAR. Flight parameters are systematically varied in order to estimate the respective effects on surveying anthropogenic terrain anomalies. External influencing factors such as different vegetation cover and seasonal acquisition time must also be taken into account as well as appropriate filter methods for DTM generation. Finally, the overall goal of this paper is to provide recommendations on how to conduct UAV-LiDAR surveys and how to filter the data to identify specific types of terrain anomalies in different types of landscapes.

## 2. Materials and Methods

### 2.1. Equipment

The system used for data acquisition consists of a RIEGL miniVUX-1UAV laser scanner device mounted on a DJI Matrice 600 drone (multicopter). The miniVUX-1UAV laser scanner operates according to the time-of-flight principle with a pulse repetition frequency of 100kHz at a near-infrared wavelength, having a laser beam divergence of 1.6×0.5mrad. At a flight altitude of for example 50m above ground level, this would lead to a laser footprint size of 8×2.5cm on the ground in nadir. Under these conditions, the system achieves an accuracy of 15mm (this value refers to the conformity of the measured value to its true value) and a precision of 10mm (this value measures how reproducible the range measurements are). The scanner is able to capture up to five returns per pulse [42].

The scanning mechanism consists of a rotating mirror that distributes the laser pulses along parallel scan lines orthogonally to the direction of flight. In order to achieve a scanning pattern as uniform as possible (i.e., the LiDAR points should be equidistant in both the cross-track and the along-track direction), the speed of the rotating mirror (LPS, lines per second) must be adjusted to the flight parameters altitude above ground and speed before each flight. The speed of the rotating mirror has no influence on the overall point density to be achieved, but only on the point pattern to be expected.

Furthermore, our RIEGL miniVUX-1UAV is equipped with a high-precision inertial measurement unit (IMU) Applanix APX-20 UAV which records roll, pitch and heading of the UAV platform at a frequency of 200 Hz with an accuracy of 0.035° with respect to heading and 0.015° with respect to roll and pitch [43].

### 2.2. Investigated Parameters

Various conditions play an important role in UAV-LiDAR data acquisition. These can be divided into flight parameters and external influencing factors.

(1) $$\mathrm{point}\;\mathrm{density}=\frac{F}{v\cdot 2h\tan(\frac{\theta }{2})}$$

**Figure 1:**
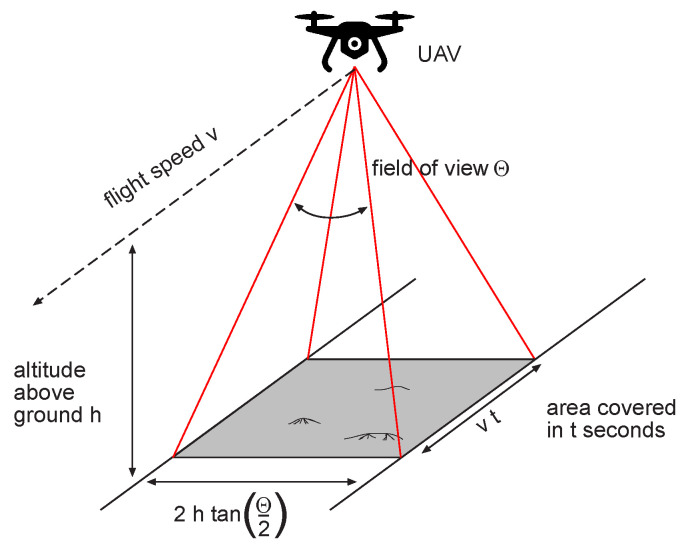
Influence of flight parameters during UAV-LiDAR data acquisition.

The LiDAR point density Equation (1), which is the central formula of this article, determines the flight parameters for data acquisition. The total number of laser shots per second is shown in the numerator, expressed by *F*, the pulse repetition frequency. The area on the ground covered by the laser system in one second is in the denominator. As Figure 1 shows, this area is calculated from the flight speed *v* and the altitude above ground h (speed multiplied by the swath width).

Thus, these two parameters are the most important factors. They are varied in this study in order to analyze their influence. The other two parameters (field of view θ and pulse repetition frequency *F*) are more specific to the scanner and cannot always be changed arbitrarily.

In the case of external influencing factors, the time of data acquisition (time of day) does not matter, since LiDAR data acquisition is independent of lighting conditions. However, the time of year when the data are collected can have a decisive influence, since vegetation cover can change during the course of a year. LiDAR is able to penetrate vegetation cover to some degree, but especially the presence of low-level vegetation in particular can be a problem when trying to separate ground from non-ground LiDAR points [35]. The term *low vegetation* (or ground level/near-ground vegetation) generally refers to vegetation with small relative height differences to their surroundings. Its definition is not entirely unambiguous. For example, Zhou et al. [35] defined growth less than 1m height as low vegetation to fit their use cases in the coastal area in south-east China. In order to provide a meaningful differentiation between tall trees and low bushes in our study areas in Germany, we decided to use the ASPRS definition for low vegetation in the context of LiDAR surveying. Following this definition according to Khosravipour et al. [44], vegetation having a relative height of less than 2m should be referred to as low vegetation.

### 2.3. Data Acquisition and Study Areas

There are several indications for the selection of suitable study areas for these analyses. Related works such as Štular et al. [40,41] distinguish different types of historical features regarding filtering and DTM generation out of ALS data. Since there has been no systematic approach to the application of UAV-LiDAR in historical conflict landscapes, we propose in this context to differentiate between the following two groups of terrain anomalies: Types of (A) historical traces and remains that show vertical differences in height from their surroundings in the order of magnitude of decimeters to meters and are therefore clearly identifiable even by on-site inspection. Examples of (A) include preserved trenches, pit structures or foxholes (dugout positions of the US Army) [26,28]. Alternatively, there are types of (B) historical traces and remains that have only very small vertical differences in height of only a few centimeters from their surroundings, making them invisible to the human eye on-site. This may be due to the fact that they are underground anomalies or have been backfilled (in the case of concave-shaped remains) or eroded (in the case of convex-shaped remains) over time. Examples of (B) include human remains and clandestine mass graves (if no burial mounds are visible on site) [45,46] or other buried historical evidence like earthwork anomalies [13] and remains of objects embedded in the ground [40].

Flight speed, altitude above ground and seasonal acquisition time/vegetation cover were examined individually in order to clarify the influence of the individual influencing factors mentioned in Section 2.2. An overview of the investigated parameters, types of anomalies and the selected study areas can be found in Table 1.

**Figure 2:**
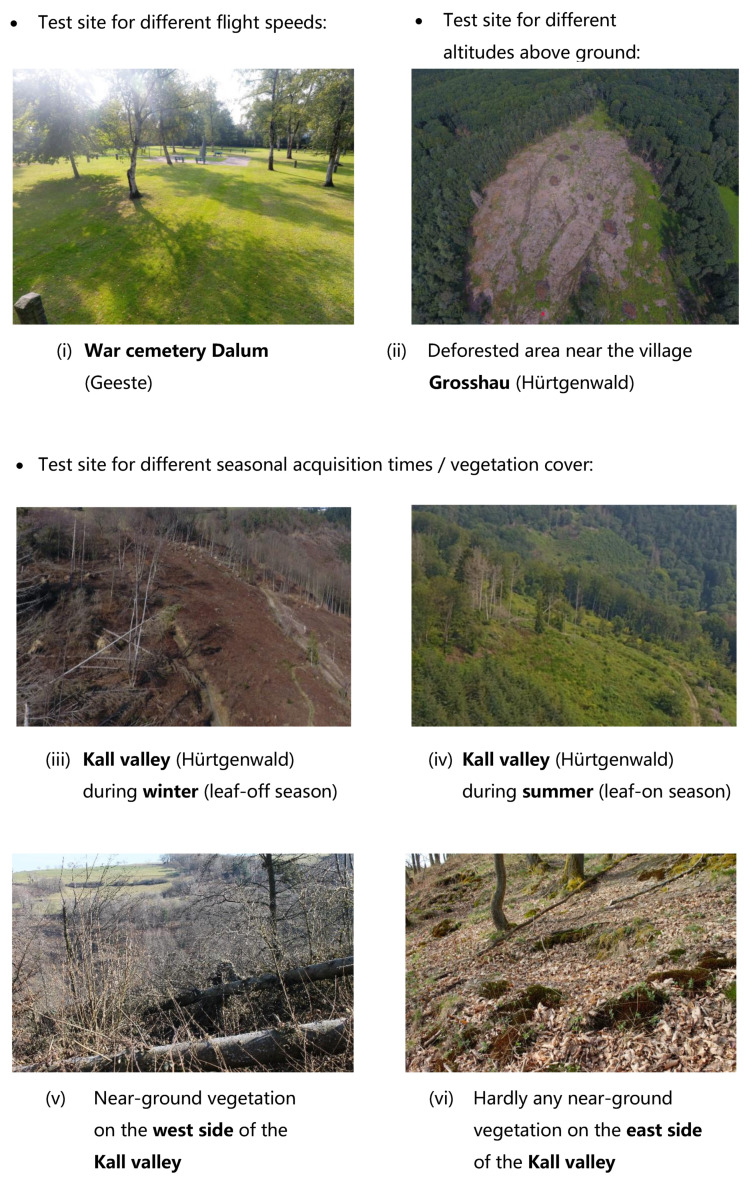
Photos of the study areas (photos taken by M. Adam, M. Storch).

Varying the *flight speed* is particularly useful when large areas of several square kilometers have to be examined. This may especially be the case if subsurface anomalies are suspected in the landscape. If their approximate positions are not known and cannot be derived from historical sources or other data, large areas must be surveyed by the UAV-LiDAR system. As a result, potentially high flight speeds are required to completely cover the survey area in a reasonable time, especially if flying higher is not an option due to even poorer spatial resolution. Therefore, we select an area with type B anomalies in order to gradually increase the flight speed and to check whether the terrain anomalies are still detectable at higher flight speeds. For terrain anomalies of this type to be recognizable at all, and for there to be no major influence from various vegetation cover (which would be a different influencing factor), the topography should be flat, and the terrain should not be heavily overgrown. This applies to the area of the *War cemetery Dalum*. The cemetery formerly belonged to “Lager XII Dalum”—one of the fifteen prisoner camps (“Emslandlager”) which existed between 1933 and 1945, situated in the region ‘Emsland’ in northern Germany. It was used for the burial and reburial presumably of foremost soviet Russian prisoners of war who had died in the ‘Emslandlager’ [47]. The cemetery today is a fenced scenically cultivated areal, approximately 120m×90m in size, overgrown with grass and surrounded by trees (see Figure 2). The landscape design includes several memorial stones, some of which are distinctively connected to certain groups of victims, but most of them seem to be more decorative although they do show the cross of the orthodox church associated with the supposed majority of the soviet Russian prisoners of war.

UAV-LiDAR data acquisition took place on 13 August 2020 at three different flight speeds, while keeping the flying altitude above ground constant. An altitude of 50m was chosen to ensure sufficient distance to the individual relatively tall trees. For the first flight, we used a flight speed of 1m/s, for the second flight, we used a flight speed of 2m/s. Then, for the third flight, we tried to cover the entire cemetery area of 1ha in less than one minute, accordingly, we chose a flight speed of 6m/s. The latter would allow for example to survey an area of 10ha in less than ten minutes under these conditions. During each flight the altitude above ground was held constant at 50m and only the speed of the rotating mirror (LPS) of the LiDAR system was adjusted in order to always achieve a uniform point pattern. It was set to 15Hz for the first flight, 25Hz for the second flight and 45Hz for the third flight.

In order to investigate the influence of different *flying altitudes above ground* it is reasonable to select an area with ground anomalies directly on the terrain surface. They are better suited to assess how well their geometry is captured by UAV-LiDAR from different altitudes than it would be the case for subsurface anomalies. Therefore, we need an area with anomalies of type A. Of course, there should be as few trees as possible in the area so that lower flight altitudes of less than 50m are possible. This applies to an area near the village *Großhau* (municipality Hürtgenwald, located in Western Germany approx. 30km south-east of Aachen near the Belgium–German border), where a trench system of the Wehrmacht was running over several kilometers and was preserved there until today when due to bark beetle infestation the area around the trench was widely deforested and therefore the ground monument was drastically destroyed. The UAV-LiDAR data acquisition took place on 4 August 2021 at six different flying altitudes above ground, keeping the flight speed constant at 2m/s. Flying altitude has been increased step by step: 10, 30, 50, 75, 100 and 120m. The latter is the currently maximum permitted flight altitude above ground for regular drone flights in most European countries, including Germany [48]. Again, only the LPS parameter was adjusted in order to generate a point pattern that is as uniform as possible every time. It was set to 50Hz for the 10m flight, 30Hz for the 30 and 50m flights, 20Hz for the 75, 100 and 120m flights.

In order to determine the effects of different *seasonal acquisition times* and *vegetation cover* on the terrain anomaly detection with UAV-LiDAR, a study area with different vegetation densities must be selected. Since no anomalies in the subsurface are expected to be revealed with such an inhomogeneous surface structure, we selected an area with type A anomalies: the *Kall valley*, which is also located in the municipality Hürtgenwald. The Kall valley was scene to violent combats between soldiers of the US Army and the Wehrmacht in November 1944 during the so called ‘battle for Schmidt’. Since it was also the area of retreat for the advancing GIs in their eventually first broken down attempt to capture the village ‘Schmidt’, it was covered with several dug out positions of the soldiers (so called ‘foxholes’) which are still detectable today. The area itself marked a challenge for the advance from the village Vossenack on the west side of the valley to Schmidt on the east side. Since the valley’s characteristics are its steep gorges and slopes, it made the crossing for tanks and other equipment almost impossible and accordingly caused the soldiers to hold their positions before any advance was possible [49,50].

The valley section considered in this study is approx. 1km long, running in a north-south direction. Thereby, this part of the Kall valley is characterized by different types of vegetation cover. The western side of the valley is dominated by low vegetation, partly predominant in summer (e.g., ferns) and partly present all year round (for example blackberry bushes). In addition, the western side of the valley is partially covered with deciduous forest, but a large part is already deforested due to drought, bark beetle infestation, storm damage and forestry work. In contrast, on the eastern side of the valley, there is a relatively intact deciduous forest with hardly any vegetation close to the ground. A relative flight altitude of 50m above ground was selected for the UAV-LiDAR survey in order to ensure sufficient distance from the trees and a permanent line of sight between the drone and the operator. A moderate flight speed of 3m/s was chosen. Due to the elongated shape of the valley, the flight planning was carried out in such a way that the flight strips were oriented perpendicular to the direction of the slope, which meant that the drone remained approximately at the same absolute altitude for each flight strip. In order to compensate for the lower point density of the downhill half of the respective upper flight strip with the higher point density of the uphill half of the lower flight strip, we tried to achieve a lateral overlap of 30% between two adjacent flight lines. The speed of the rotating mirror was set to 30Hz. The UAV-LiDAR data acquisition took place on 19/20 August 2020 under leaf-on conditions and on 6 March/1 April 2021 under leaf-off conditions.

### 2.4. Data Processing

The LiDAR data processing always begins with the necessary step of converting the raw range measurements recorded by the laser scanner into point cloud data. To do this, the flight trajectory which results from the drone’s IMU motion measurements must first be corrected using the GNSS correction data acquired by the GNSS base station. These GNSS observation data (so-called observables) were imported into the Applanix POSPac software via the RINEX file format together with the IMU data in order to correct the raw flight trajectory. The resulting SBET (Smoothed Best Estimate of Trajectory) was then further processed within RIEGL’s RiPROCESS software which includes the combination of the raw scan data with the SBET information and the global registration in order to generate geo-referenced point clouds with UTM-coordinates. Additional efforts in across-flight line registration were made by the automatic search of tie-planes using RIEGL’s RiPRECISION plugin within RiPROCESS. Finally, the point clouds were exported to the LAS file format. This standard processing workflow is outlined in a more detailed way in, e.g., Brede et al. [51] or Ten Harkel et al. [52].

A crucial step in the processing of LiDAR data and the creation of terrain models is the determination of which LiDAR points represent objects near the ground surface and which represent the ground surface itself. Only the latter will later serve as input into the calculation of a DTM. This process of separating LiDAR data into ground and non-ground data is called filtering, and there are a variety of such algorithms, each of which may be suitable for different types of landscape.

The relief and environmental conditions in a landscape such as the *Dalum cemetery* or the trench near *Großhau* (flat, open terrain with single trees) do not normally pose a challenge for filtering algorithms [37]. A frequently used algorithm is the Cloth Simulation Filter Algorithm (CSF) proposed by Zhang et al. [53]. The principle of CSF is to simulate a rigid cloth that is placed on top of the inverted point cloud. The cloth has a user-defined rigidness, and its shape gradually adapts to the inverted LiDAR data due to the simulated gravity. The final shape of the cloth then serves as a reference to divide the original LiDAR points into ground and non-ground points. According to Chen et al. [39], CSF therefore belongs to the category of surface-based methods which begin with the creation of an initial surface and refine it iteratively towards the final selection of ground points. It provides good results when filtering UAV-based point clouds, especially in flat, non-complex terrain situations [54]. Hence, we used the CSF method to filter the point clouds from the *Dalum cemetery* and *Großhau*.

In contrast to the other study areas, the *Kall valley* is characterized by a complex terrain with varying vegetation densities, a lot of low vegetation and steep gradients (see Section 2.3), which is a challenge for many filter algorithms [35,37]. Although CSF is generally regarded as a very robust filtering method [54], it can be unsuitable for this type of landscape [55]. Hence, we decided to choose the Simple Morphological Filter (SMRF) [56] as a second filter method which follows a completely different approach than CSF. SMRF is a further development of the progressive morphological filter [57,58] and thus belongs to the group of morphology-based methods according to the algorithm classification scheme developed by Chen et al. [39]. SMRF is based on a structuring element with gradually increasing neighborhood size that performs opening operations (erosion followed by dilation) on the point cloud. In each iteration, an elevation threshold is calculated based on the user-defined slope tolerance parameter which is then applied to the opened surface to distinguish between ground and objects [56]. However, the applicability of morphology-based methods like SMRF for this type of terrain is to some extent controversial [39,55,59] so that we additionally applied a third filter algorithm. Since TIN-based methods are considered to be well-suited for steep terrain situations [39], we use such an algorithm as a third variant: the Adaptive Triangulated Irregular Network approach (ATIN) which was originally proposed by Axelsson [60]. It starts by creating an initial TIN from a set of seed points that are determined from a grid with user-defined cell size, using the lowest points per cell. Then, each point is iteratively checked and marked as ground if its distance to the ground TIN and angles to the nodes do not exceed a certain threshold value. As a final denoising step, we applied the *feature preserving smoothing algorithm* proposed by Lindsay et al. [61] to the rasterized ground point data from the Kall valley, which is a DTM noise removal algorithm based on the modification of surface normals.

Subsequently, suitable visualization methods must be selected for all use cases to facilitate the visual interpretation of the DTMs. Extensive research has already been carried out on this topic in several studies [62,63,64,65,66,67,68]. We opted for the multidirectional hillshading technique because a shaded relief generally appears to be the most natural and intuitive product for visualization. In addition, compared to unidirectional hillshading with only one light source, it has the advantage that elongated anomalies can be detected regardless of their direction [67,68].

## 3. Results

### 3.1. Influence of Flight Speed

UAV-LiDAR data were collected from the cemetery site at three different flight speeds (see Section 2.3). After pre-processing, filtering was performed using CSF. The filter is parameterized according to the recommendations of, e.g., Zhang et al. [53] and Serifoglu Yilmaz et al. [54]. The maximum number of iterations is set to 500, time step size is set to 0.65 and rigidness is set to 3 for flat terrain. The cloth resolution is set to 0.2m to account for the high resolution input data, and the classification threshold is set to 0.1m to ensure that even small memorial stones with a height of a few decimeters located on the surface are not classified as ground. The metadata of the resulting ground point clouds are summarized in Table 2.

It should be noted that the actual received return density is higher than the laser shot density, as a single emitted laser shot can be returned multiple times, e.g., by the vegetation canopy. Doubling the flight speed from 1m/s to 2m/s reduces the laser shot density by 50%. It has a similar effect on the ground point density and leads to an increase in the average ground point spacing from 7cm to 10cm. A tripling of the flight speed from 2m/s to 6m/s reduces the density values further by 2/3 and leads to an average ground point spacing of 17cm. This was to be expected, because the LiDAR point density Equation (1) denotes an inverse relationship between flight speed and resulting point density.

**Figure 3:**
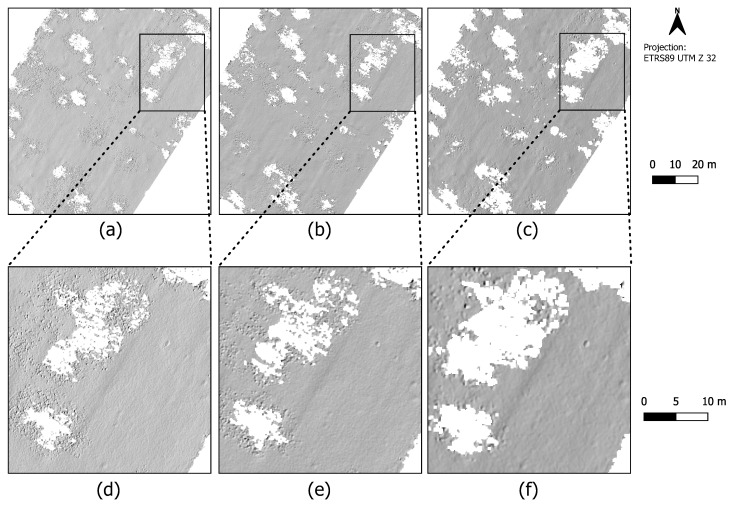
Multi-directional hillshade results from the filtered UAV-LiDAR scans of the Dalum cemetery site. Upper row: (**a**) flight at 1m/s, (**b**) flight at 2m/s, (**c**) flight at 6m/s. Bottom row: (**d**–**f**) data cutout of each (**a**–**c**) at larger scale.

Figure 3 shows the multi-directional hillshade representations of the point clouds which were recorded at three different flight speeds (a–c), each filtered by using the CSF method. Illustrations (d–f) show an exemplary data excerpt from the three data sets on a larger scale. The spatial resolution of each grid results from the respective mean horizontal ground point spacing. The minimum height value is assigned to each cell. Especially in the high resolution point clouds (a) and (b) resulting from the slower speed flights, elongated anomalies in the terrain surface that are oriented from southwest to northeast are noticeable. These anomalies are also visible in the high-speed flight data (c) when looking at the treeless open terrain. However, it is noticeable that the extent of the areas without ground points, i.e., without ground information (marked in white in Figure 3), is significantly greater in the data from the 6m/s flight than in the data from two lower speed flights. These are areas where the data acquisition is disturbed due to several trees in the open space. Consequently, no statement can be made about the presence/course of the terrain anomalies in these particular areas.

### 3.2. Influence of Altitude above Ground

UAV-LiDAR data were collected from the trench near the village of Großhau at different flight altitudes above ground (see Section 2.3). The CSF ground filter is applied in a similar way to Section 3.1. The metadata are summarized in Table 3.

As with the flight speed, the LiDAR point density Equation (1) also denotes an inverse relationship between point density and altitude above ground. Higher flight altitudes lead to lower point densities. For example, a tenfold increase in flight altitude from 10 m to 100 m leads to a reduction in the point density to approx. 1/10 from 727 points/m^2^ to 69 points/m^2^.

**Figure 4:**
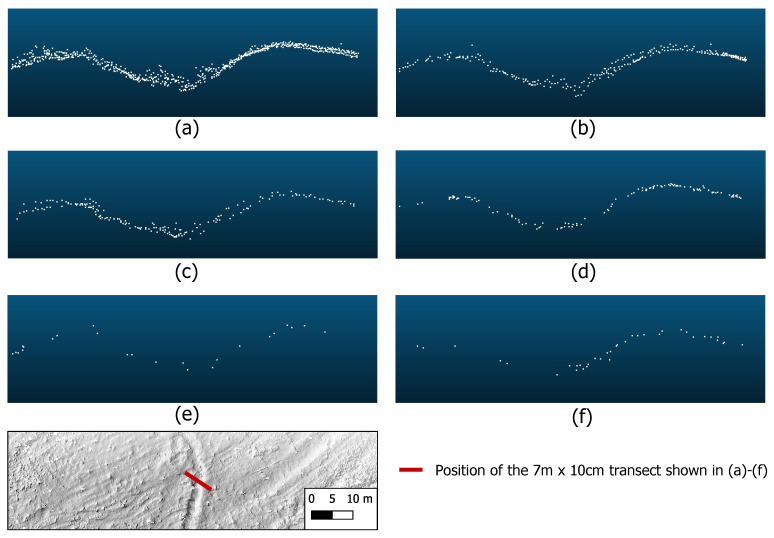
Side view of a 7m×10cm transect through each point cloud of the trench near Großhau (Hürtgenwald), acquired at different flight altitudes above ground: (**a**) flight at 10m, (**b**) flight at 30m, (**c**) flight at 50m, (**d**) flight at 75m, (**e**) flight at 100m, (**f**) flight at 120m.

To illustrate the difference in how detailed the shape of the trench was recorded, Figure 4 shows a transect through each point cloud.

The rough shape of the trench was correctly captured from all tested flight altitudes. Of course, the increase in average point spacing with increasing altitude results in a less detailed surface. While at an altitude of 10 m with a point spacing of 4 cm even the smallest structures within the trench (e.g., deadwood, small shrubs, etc.) can be captured, only a much smoother surface is recorded in the data from for example 75, 100 or 120 m altitude above ground, with an average ground point distance of only 12, 14 or 15 cm. Since the ditch has a total width of several meters, its general shape is detectable even in the data resulting from the highest altitude flight, although some of the LiDAR points are very far apart. In the example data excerpt shown in Figure 4, the maximum horizontal distances between two neighboring points on the transect are 76 cm for the 120 m flight and 52 cm for the 100 m flight (see Table 3). Looking at the 75 m flight, the maximum horizontal distance between two neighboring points along the transect is reduced to 28 cm, which is almost half as much as in the data of the 100 m flight and almost a third as much as in the data of the 120 m flight. If the flight altitude is further reduced to 50 m, the maximum point distance has been more than halved compared to the 75 m flight and is only 13 cm.

**Figure 5:**
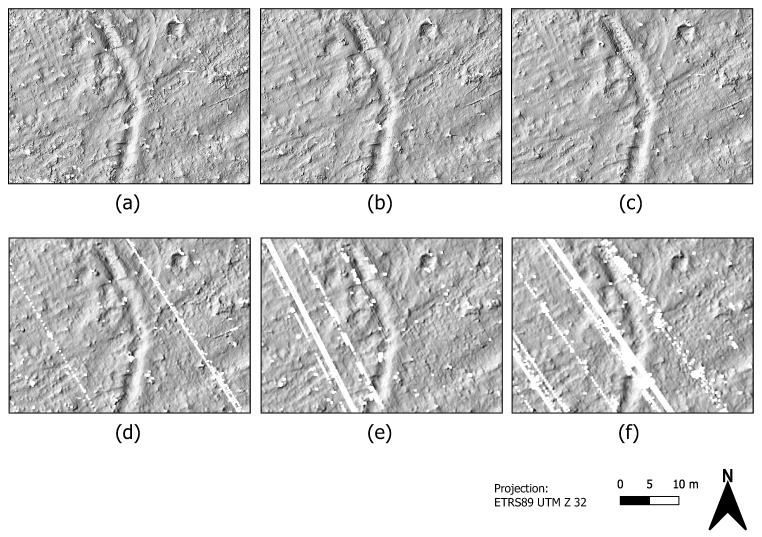
Multi-directional hillshade results from the UAV-LiDAR scans of the trench near Großhau (Hürtgenwald), acquired at different flight altitudes above ground: (**a**) flight at 10m, (**b**) flight at 30m, (**c**) flight at 50m, (**d**) flight at 75m, (**e**) flight at 100m, (**f**) flight at 120m.

Figure 5 shows the multi-directional hillshade representations of the point clouds of the trench, which were recorded at different flight altitudes above ground.

Analogous to the procedure described in Section 3.1, the spatial resolution of each grid is again determined from the respective mean horizontal ground point spacing and the minimum height value is assigned to each cell. It becomes clear that the trench was captured in the data of all tested flight altitudes since it is visible in every hillshade image. At this zoom level, no major differences between the hillshade images from the 10m, 30m and 50m flights are visually discernible. Above a height of 75m, however, larger gaps become apparent in the data. Since the flight path ran from southwest to northeast, these are likely due to the fact that at higher flight altitudes the distance between two consecutive scan lines increases. At altitudes over 100m above ground and a correspondingly low LPS value (we set LPS to 20Hz for these flights), two consecutive scan lines are more than 10cm apart. Under these circumstances, if the drone pitches briefly due to a gust of wind, for example, which is not unlikely at these flight altitudes, even larger scan line distances can quickly arise, which explains the data gaps.

However, there is another important factor to consider: the diameter of the laser footprints on the ground, which is a function of the flight altitude and the laser beam divergence (see Table 3). It increases gradually from 1.6cm×0.5cm at 10m altitude to 19cm×6cm at 120m altitude above ground. Objects or details of the trench shape that are smaller than the respective laser beam footprint cannot be captured by the UAV-LiDAR.

### 3.3. Influence of Seasonal Acquisition Time and Vegetation Cover

The relief and environmental conditions in the Kall valley consist of steep slopes and different vegetation density. As described in Section 2.4, we tried three different algorithms, each belonging to a different class of methods. CSF is parameterized in a similar way to the other use cases, with the exception for the rigidness parameter which is set to 1 in order to take into account the steep terrain relief. SMRF is parameterized as follows: According to Pingel et al. [56], the window radius should not be smaller than 10m, and the slope tolerance should not be lower than 10%. The use of these values together with a cell size of 0.2m similar to the selected CSF cloth resolution results in a maximum elevation threshold of 1m. Since foxholes can have vertical extents of this order of magnitude, this value seems reasonable in order not to filter out the terrain anomalies. The final classification threshold is then set to 0.1m similar to the CSF parameterization. In order to parameterize the ATIN filter algorithm, Klápště et al. [55] suggest not to set the cell size for the initial user defined grid below 25m. This value should furthermore correspond to the largest existing non-ground object [54]. In our study area, this roughly corresponds to isolated groups of trees with dense foliage so that the parameter value mentioned above is justified. The maximum angle parameter should be selected according to the average inclination of the area [54], which in our case is about 20° (the approximate value was derived from the DTM generated from the SMRF result). Furthermore, we set the parameter for the maximum distance to 1m similar to the maximum elevation threshold of SMRF.

**Figure 6:**
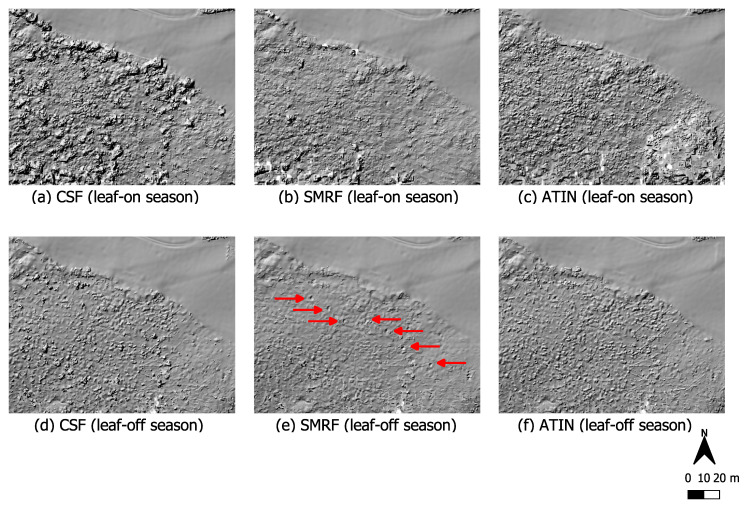
Data cutout of a deciduous forest covered section on the western Kall valley. Near-ground vegetation is present here throughout the whole year (see photo (v) in Figure 2). Data acquisition during summer (upper row) and winter (bottom row). Point clouds were filtered using three different filter algorithms, CSF, SMRF and ATIN.

First, we present the UAV-LiDAR data that were obtained on the western side of the Kall valley. Figure 6 shows a section with relatively complete deciduous forest. In addition, there is strong ground overgrowth with low vegetation in the form of bushes, for example blackberry bushes, all year round (see photo (v) in Figure 2). In summer, when both ground level vegetation and tree foliage are fully developed, the terrain anomalies are not visible in the data. Many LiDAR points representing vegetation are obviously misclassified as ground; however, SMRF apparently provides the smoothest result with less ground level noise compared to the other two.

Looking at the winter situation in which the leaves of the deciduous trees are not yet developed at this time of year, it becomes apparent that terrain anomalies only become visible in the data when using SMRF as a filter method (circular depressions lined up in a row from north-west to south east). Table 4 summarizes the calculated point densities and point spacings of the ground filter results. With the winter data, the resulting point density is almost twice as high as with the summer data. The mean point spacing is reduced from 11–13cm to 8–9cm.

**Figure 7:**
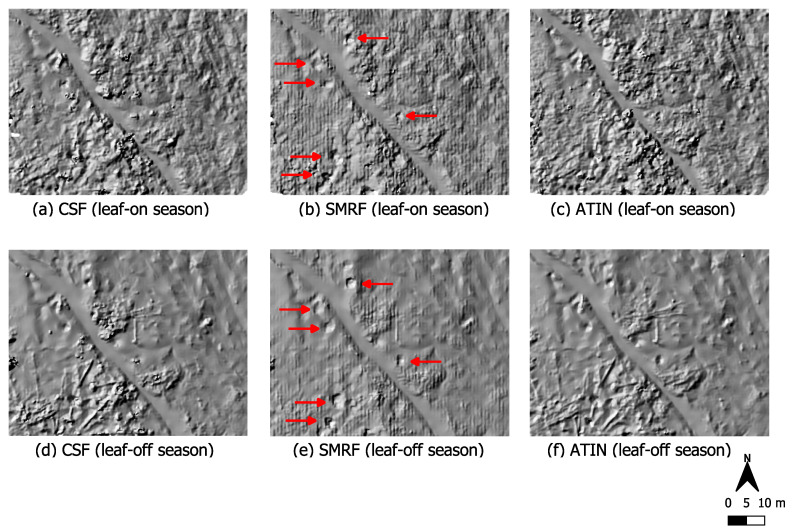
Data cutout of a predominantly deforested section on the western Kall valley. Near-ground vegetation is present here mainly during summer (see photos (iii) and (iv) in Figure 2). Data acquisition during summer (upper row) and winter (bottom row). Point clouds were filtered using three different filter algorithms, CSF, SMRF and ATIN.

Figure 7 shows a different part of the western Kall valley, which is largely deforested (see photos (iii) and (iv) in Figure 2). In winter, the ground vegetation is not very pronounced, and in summer, low vegetation by ferns, etc., predominates. Here, it can be seen that SMRF again delivers the visually smoothest results. This filter can obviously filter out more of the low vegetation that is present here in summer than the other two methods. In (b), some circular depressions are at least barely visible, while in (a) and (c), they can hardly be identified at all. The situation is similar with regard to the winter data. At (d) and (f), you can even see deadwood, which was apparently incorrectly classified as ground by the filter algorithms CSF and ATIN but was successfully removed by SMRF, which makes the terrain anomalies stand out better here (e).

However, the SMRF method apparently introduced noise into the resulting ground point data. The difference between summer and winter in terms of the resulting point densities is smaller than in the area shown previously: ground point densities range from 137–203 points per square meter for the summer data and 150–189 points per square meter for the winter data. The point spacing is between 8–9cm.

**Figure 8:**
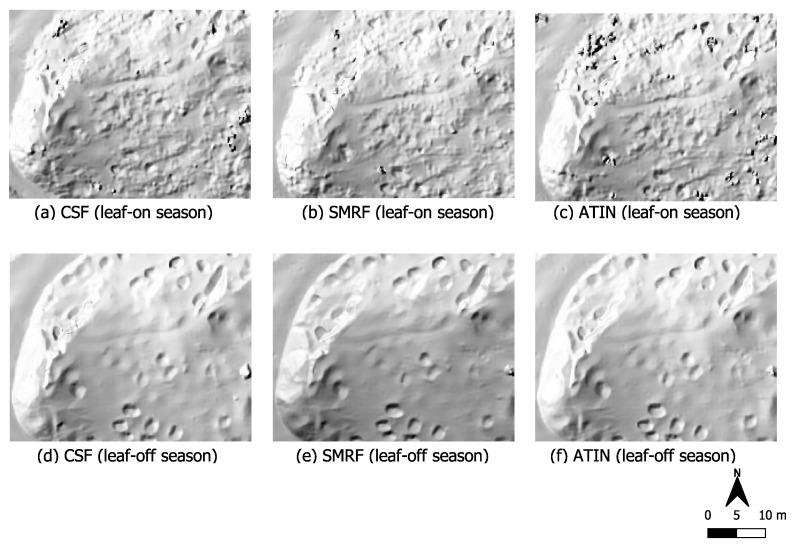
Data cutout of a deciduous forest covered section on the eastern Kall valley. Hardly any near-ground vegetation is present here the whole year round (see photo (vi) in Figure 2). Data acquisition during summer (upper row) and winter (bottom row). Point clouds were filtered using three different filter algorithms, CSF, SMRF and ATIN.

In order to clarify the influence of deciduous forest stands with almost complete absence of vegetation close to the ground on the surveying of such terrain anomalies, we now compare these data from the western side of the Kall valley with the data from the eastern side of the valley. As described in Section 2.3, the vegetation in this part of the eastern Kall valley in contrast to the western side of the valley consists only of deciduous trees and hardly any near-ground vegetation (see photo (vi) in Figure 2). In contrast to the sections shown before, the results of the three filter algorithms here look very similar to each other, almost identical (see Figure 8). This is underlined by the metadata in Table 4, in particular for the data acquired under leaf-off conditions. All three filter methods used each produce a ground point cloud with an average point spacing of 9cm, and point densities range between 121 and 128 points per square meter. This differs from the summer situation when there is obviously less ground information in the data. The average point density is only half that of the winter data or sometimes even less. The mean point spacing is between 13–15cm.

In the hillshade image of the summer data, the circular depressions in the ground are more difficult to identify. Especially in the north-western part of the section along the foot path, they do not seem to have been recorded at all in the summer data. This difference is caused solely by the emergence of the foliage on the trees, as there is no low vegetation in this area all year round.

## 4. Discussion

Through our analyses, we systematically illustrate the influence of various factors on UAV-LiDAR data acquisition within the scope of terrain anomaly surveying in historical conflict landscapes.

Regarding the *flight speed*, which we varied on the terrain of the War cemetery Dalum, it can be said that this–at least in our chosen range of speeds–apparently plays a rather subordinate role. The underground terrain anomalies were evident in the data from all three flight speeds tested. They were also visible in the flight at the highest tested speed, but here, the mean horizontal ground point spacing is relatively high at 17cm. This does not appear to affect the ability to visually identify these ground anomalies in the hillshade image of the open terrain. The decisive factor here is that although the point density decreases with increasing flight speed and thus the average point spacing increases, the diameter of the laser pulses on the ground (laser footprint size), which plays an important role in how accurate the terrain surface can be captured (in the horizontal and vertical direction) [14], is not directly related to this. The size of the laser footprint is more influenced by the flight altitude and the slope of the terrain. Thus, even at higher flight speeds, despite an overall lower point density, small height differences in the terrain can be detected by the UAV-LiDAR system and finally recognized in the resulting hillshade image of the DTM generated from the LiDAR data.

For the 6m/s flight in particular, however, it has been shown that in the case of individual trees on the site, due to the complete lack of ground points, no statement can be made about the presence or course of the terrain anomalies in these areas. This means that the choice of flight speed is particularly important if the area to be surveyed is not entirely free of trees/vegetation. In this case, slower speeds like 1m/s or 2m/s should therefore be selected in order not to risk losing valuable ground information in the immediate vicinity of the trees. In addition, ground-level measurements, e.g., with ground penetrating radar, could be useful here to obtain further information about the terrain anomalies, together with GNSS or total station measurements in order to locate them precisely [31,33].

On the other hand, a higher speed such as the tested 6m/s can be selected without hesitation in open terrain. A flight speed of this order of magnitude would make it possible to cover an area of 10ha in less than ten minutes under these conditions. In these cases, it is therefore possible to quickly gain an overview of where terrain anomalies are located in the landscape.

The anomalies found then might be a reference for further interdisciplinary investigation on the ground as well as another indication of what might have happened in the area in the past—although they are hardly clear evidence since ‘conflict landscapes’ are known to be heavily transformed in the time after their emergence/development.

With regard to the *altitude above ground*, we tested six different altitudes in a study area with a terrain anomaly visible on the surface of the earth, a Second World War trench. We illustrated the differences in the level of detail on a 7m×10cm transect through the point clouds. The possible uses of these point clouds depend on the intention with which the data are being collected. If it is to be examined whether there is vegetation, deadwood or other smaller objects in the trench, a dense point cloud is needed. With a mean ground point spacing of 4, 6 and 8cm, the point cloud data from the 10, 30 and 50m would be suitable for these purposes. However, since filtering of such low objects/vegetation is not a trivial task [40], so much detail can be more distracting than useful. For historians it generally is more relevant to record for example the shape of the ditch shoulders in order to find out about the origin/purpose of such anomalies, on the one hand, and assess the erosion state of the historical remains, on the other hand. Since the data resulting from the 75m flight represents a much smoother surface in the first place, they are therefore more suitable for these purposes than the point clouds from the lower altitude flights. The reason for this is the larger footprint size. As also confirmed by the work of Fernandez-Diaz et al. [14], larger footprints cover a larger area on the ground per pulse so that the backscattered signal is a mixed signal of the covered elevations. On the one hand, this leads to a more uniform return pattern in the data from the flights at higher altitudes, but on the other hand, it has the disadvantage of lower horizontal and vertical accuracy.

It is also noticeable that larger gaps in the data occur at the higher flight altitudes, especially at 100 and 120m above ground. The maximum horizontal distance between two neighboring points on the selected transect is 52cm for the 100m flight and 76cm for the 120m flight. At such altitudes above ground, the UAV is naturally susceptible to gusts of wind which can result in pitch moves (tilting along the transverse axis). This can lead to larger distances between consecutive scan lines so that such strip-shaped data gaps can then occur. In the worst case, there is a risk that smaller anomalies will not be detected. However, sometimes topography or high vegetation, for example, can make it hard to comply with VLOS (visual line of sight). In most European countries, including Germany, there must be direct visual contact between the operator and the UAV at all times for regular drone flights, which could mean that high flight altitudes like 100 or 120m must be chosen. In the case of elongated anomalies, we therefore recommend flying in such a way that the scan lines are arranged orthogonally to the terrain anomaly. This means that if a laser scanner like our RIEGL miniVUX-1UAV is used which operates with a rotating mirror that distributes the laser pulses along parallel scan lines orthogonal to the direction of flight, then the direction of flight should be aligned parallel to the anomaly. However, this does not apply to LiDAR systems with different scanning mechanisms, for example, Palmer scanners that generate elliptical point patterns [14].

In this context, it is also important to consider what added value these data might have for maps or other cartographic products. Usually, for this type of terrain anomaly, it is important to create illustrations to document for example the course of such a ditch in forested areas or a complete ditch system (e.g., a sky-view factor, local relief model or hillshade image of the DTM [65,66,67,68]). On the one hand, this would not require such a high resolution of 4cm as resulting from the 10m flight, and at such a flight altitude, it would take a long time to survey larger areas. In a forested area, such a low altitude would not be feasible anyway. On the other hand, the data must not have such large gaps of more than half a meter as resulting from the 100 and 120m flights so that the trench system would no longer be recognizable and thus the resulting map illustrations would no longer be useful for interpretation. Thus, it can be seen that even from a cartographic point of view, an average flight altitude in the order of 50–75 m proves to be suitable by providing a good compromise.

In order to clarify the influence of *seasonal acquisition time* and *vegetation cover*, we chose the historical conflict landscape of the Kall valley as the study area. Data acquisition was carried out during winter under leaf-off and summer under leaf-on conditions in different parts of the valley. We used three filter algorithms to separate ground from non-ground LiDAR points, each as a representative of a different class of methods.

With regard to vegetation cover, it can be seen that in areas with a lot of near-ground vegetation, which is also present during winter in some parts of the Kall valley, the morphological filter SMRF provides the smoothest filter results (Figure 6 and Figure 7). This coincides to a certain extent with Klápště et al. [55] who consider the morphological filter to be suitable for this type of landscape, while ATIN and CSF provided poorer results in their study as well. These filters apparently cope worse with the existing ground vegetation. Especially ATIN can be problematic with much low vegetation like bushes [54]. The calculated point densities make clear that ATIN in particular has higher densities of resulting ground points compared to the other filtering methods used. As summarized in Table 4, ATIN produced on average 25% higher point densities than the other two filters when focusing on areas where low vegetation is present. Thus, the determined point spacing is approx. 1–2cm lower than with the other methods. However, more resulting points do not mean better detection of the ground anomalies because here these are more often incorrectly classified points. Objects such as dead wood were classified as soil (false positives). While this does not make it impossible to identify the terrain anomalies in each area, it does make it more difficult and severely limits the suitability of these data for historical interpretation purposes, e.g., to detect anomalies which indicate the form of typical dug out positions. Based on the results of our experiments, we therefore recommend the use of a morphological filter such as SMRF to reveal terrain anomalies in areas with much low vegetation. This confirms, for example, the work of Chen et al. [39] and Mongus et al. [59] who stated that morphology-based filters are well-suited to remove small non-ground objects.

For our data excerpt in Figure 7, which shows a widely deforested area, this applies to both the winter data and the summer data. Defoliated trees only play a subordinate role here. On the contrary, in the part of the Kall valley which is shown in Figure 6, not even SMRF could make the terrain anomalies visible when considering the data obtained under leaf-on conditions. In summer, this is an area with very pronounced vegetation close to the ground together with leafy, intact deciduous tree population. Accordingly, if both intact forest and ground level vegetation come together, such areas should only be surveyed under leaf-off conditions in order to obtain sufficient ground information for the survey of terrain anomalies.

In an area with an only deciduous forest stand (Figure 8), again a clear difference between leaf-off and leaf-on conditions could be seen. In the data acquired during winter season under defoliated conditions, all three filters produce a very smooth ground surface, which makes the circular terrain anomalies stand out strongly. Obviously less ground information is included in the data in summer. The average point density is only half that of the winter data or even less. This reflects the results of the area depicted in Figure 6. However, in contrast to that area, there is hardly any near-ground vegetation present in Figure 8. This circumstance leads to the fact that all three filters deliver almost identical filter results, especially with regard to the winter situation where the resulting ground point densities of the three algorithms have a percentage deviation of less than 6% from each other. We infer from these results that in the complete absence of ground level vegetation, the choice of the filter algorithm plays a subordinate role. For our data, the surface-based, the morphological-based as well as the TIN-based filter provided all results suitable for the detection of terrain anomalies and historical interpretation purposes.

Table 5 summarizes the recommendations for the use of UAV-LiDAR in historical conflict landscapes based on the results of our experiments:

The interdependence of the individual influencing factors must be taken into account. For example, in an area where subsurface anomalies are suspected, the density of tree cover plays a role in choosing the appropriate flight speed. In addition, with regard to the altitude above ground, it has been found that if the altitude is too high (more than 75m above ground) data gaps occur due to pitch movements of the UAV, which are presumably caused by gusts of wind. If such an altitude were unavoidable for whatever reason, a possible solution—in addition to the above-mentioned distribution of the laser scan lines orthogonal to the terrain anomaly—could also be to choose a lower flight speed than the 2m/s we selected in the altitude experiments.

The two remaining parameters *field of view* and *pulse repetition frequency* were not investigated in this study. We did not consider these parameters to be universal as they are rather scanner-specific and cannot always be changed arbitrarily for every UAV-LiDAR scanner system. With regard to the pulse repetition frequency, the following applies: the lower this value, the lower the laser shot density, but at the same time, the emitted energy is distributed over fewer pulses. This can lead to a higher canopy penetration so that more laser pulses reach the ground in vegetated areas [14,35].

Further research could deal with improvements in the filtering of low vegetation in order to further reveal and highlight the terrain anomalies in the DTMs. Ground-level measurements such as total station surveys could be used to determine the absolute accuracy with which the ground surface was recorded. In addition, the LiDAR intensity attribute could provide additional benefit in detecting anomalies in the subsurface, since the return intensity depends to some extent on, for example, soil moisture [69]. Furthermore, a systematic combination of UAV-LiDAR with other remote sensing technologies, for example, multispectral, hyperspectral or thermal sensors, is conceivable in order to detect cropmark anomalies caused by historical remains in the subsurface, as some recent other studies indicate [13,70]. However, the data recorded by passive optical-reflective remote sensors are affected by external environmental factors such as the lighting conditions, which could to some extent be a challenge for data fusion approaches.

## 5. Conclusions

Through various drone flights in a wide variety of conflict landscapes, we have investigated the influence of different factors on UAV-LiDAR data acquisition for surveying anthropogenic terrain anomalies in historically relevant landscapes. The flight parameters speed and altitude above ground were systematically varied. It was shown that for the detection of anomalies below the surface in treeless open terrain, higher flight speeds such as 6m/s are feasible. With regard to the flight altitude, we recommend an altitude of 50–75m above ground for detecting surface structures with low noise in the data. We also examined the influence of vegetation cover in relation to the seasonal acquisition time. Areas covered with deciduous forest should only be surveyed in winter during leaf-off season. In the presence of low-level vegetation, it can be seen that data acquisition in summer provides less ground information than in winter, but it is still possible to obtain information on existing terrain anomalies. In our study, the morphological filter SMRF proved to be best suited to filter out such near-ground vegetation. However, in complete absence of low vegetation, the choice of the filter algorithm plays a subordinate role. Further research could deal with improvements in the filtering of low vegetation in order to further reveal the terrain anomalies in the DTMs, supported by reference measurements in the field especially in heavily overgrown areas. In addition, the additional use of the LiDAR intensity attribute and a combination of UAV-LiDAR with other remote sensing technologies, for example, multispectral, hyperspectral or thermal sensors, is conceivable.

## Figures and Tables

**Table 1:** Overview of the investigated parameters, the types of anomalies, the requirements and the selected study areas.

Investigated Parameters	Type of Anomalies	Topography and Vegetation	Study Area
Flight speed	Type B	flat, open terrain	War cemetery Dalum (municipality Geeste)
Altitude above ground	Type A	open terrain	Trench near Großhau (municipality Hürtgenwald)
Acquisition time (seasonal), vegetation cover	Type A	different density of high and low seasonal vegetation	Kall valley (municipality Hürtgenwald)

**Table 2:** Metadata of the unfiltered and filtered point cloud data (War Cemetery Dalum) acquired at three different flight speeds, keeping the flying altitude constant at 50 m above ground.

	Flight Speed	1 m/s	2 m/s	6 m/s
unfiltered	Mean laser shot density (last returns)	295 pulses/m^2^	152 pulses/m^2^	49 pulses/m^2^
	Mean horizontal laser shot spacing (last returns)	6 cm	8 cm	14 cm
	Mean point density	412 points/m^2^	215 points/m^2^	69 points/m^2^
	Mean horizontal point spacing	5 cm	7 cm	12 cm
	Mean ground point density	206 points/m^2^	104 points/m^2^	33 points/m^2^
	Mean horizontal ground point spacing	7 cm	10 cm	17 cm

**Table 3:** Metadata of the unfiltered and filtered point cloud data (trench near Großhau) acquired at different flight altitudes above ground, keeping the flight speed constant at 2 m/s.

	Altitude above Ground	10 m	30 m	50 m	75 m	100 m	120 m
unfiltered	Mean laser shot density (last returns) [pulses/m^2^]	727	282	164	81	65	54
	Mean horizontal laser shot spacing (last returns)	4 cm	6 cm	8 cm	11 cm	12 cm	14 cm
	Mean point density [points/m^2^]	727	287	175	86	69	57
	Mean horizontal point spacing	4 cm	6 cm	8 cm	11 cm	12 cm	13 cm
	Mean ground point density [points/m^2^]	672	253	138	71	54	44
	Mean horizontal ground point spacing	4 cm	6 cm	8 cm	12 cm	14 cm	15 cm
	Maximum horizontal distance between two adjacent points along the transect shown in Figure 4	<10 cm	11 cm	13 cm	28 cm	52 cm	76 cm
	Laser beam footprint diameter on the ground in nadir [cm × cm]	1.6 × 0.5	4.8 × 1.5	8 × 2.5	12 × 3.75	16 × 5	19 × 6

**Table 4:** Metadata of the filtered point cloud data acquired in different areas of the Kall valley with varying vegetation cover, acquired during leaf-on (summer) and leaf-off (winter) conditions.

	Area Covered with		Results of Ground Filter	(Summer Data)			(Winter Data)		
	Low veg.	dec. Forest		CSF	SMRF	ATIN	CSF	SMRF	ATIN
Figure 6	√	√	Mean point density [pts/m^2^]	70	63	84	122	117	144
			Mean point spacing	12 cm	13 cm	11 cm	9 cm	9 cm	8 cm
Figure 7	√		Mean point density [pts/m^2^]	165	137	203	171	150	189
			Mean point spacing	8 cm	9 cm	7 cm	8 cm	8 cm	7 cm
Figure 8		√	Mean point density [pts/m^2^]	55	47	61	122	128	121
			Mean point spacing	14 cm	15 cm	13 cm	9 cm	9 cm	9 cm

**Table 5:** Summary of recommendations to be made for the use of UAV-LiDAR in historical conflict landscapes based on our research conducted. (Reminder: the term *type A* anomalies refers to anomalies located on the earth’s surface, and the term *type B* anomalies refers to subsurface anomalies; see Section 2.3).

Influencing Factor	Objective/Research Area		Recommendations
Flight speed	Type B anomalies in partially vegetated area		Lower speeds in the range of 1–2 m/s
	Type B anomalies in treeless open terrain		Higher speeds like 6 m/s are justifiable
Altitude above ground	Type A anomalies. Capture the ground with		Higher flying altitudes in the range
	as little noise as possible caused by low objects		of 50–75 m
Vegetation cover	**Near-Ground Vegetation**	**Deciduous Forest**	
	√	√	Data acquisition during winter (leaf-off),
			use morphological filter, e.g., SMRF
	√		Data acq. during summer or winter,
			use morphological filter, e.g., SMRF
		√	Data acquisition during winter (leaf-off),
			filter method plays a subordinate role

## Data Availability

Data are contained within the article.
